# Prognostic value of a decrease in mean platelet volume, platelet distribution width, and platelet-large cell ratio for major adverse cardiovascular events after myocardial infarction without ST-segment elevation: An observational study

**DOI:** 10.17305/bb.2023.9178

**Published:** 2023-10-01

**Authors:** Emir Bećirović, Kenana Ljuca, Minela Bećirović, Nadina Ljuca, Mugdim Bajrić, Ammar Brkić, Farid Ljuca

**Affiliations:** 1 Internal Medicine Clinic, University Clinical Center Tuzla, Tuzla, Bosnia and Herzegovina; 2 School of Medicine, University of Tuzla, Tuzla, Bosnia and Herzegovina; 3 Health Center of Sarajevo Canton, Sarajevo, Bosnia and Herzegovina; 4 Department of Cardiovascular Surgery, University Clinical Center Tuzla, Tuzla, Bosnia and Herzegovina; 5 Department of Physiology, School of Medicine, University of Tuzla, Tuzla, Bosnia and Herzegovina

**Keywords:** Mean platelet volume (MPV), platelet distribution width (PDW), platelet-large cell ratio (P-LCR), major adverse cardiovascular events (MACEs), myocardial infarction without ST-segment elevation (NSTEMI)

## Abstract

The current study aimed to explore whether the level of decrease in platelet distribution width (PDW), platelet-large cell ratio (P-LCR), and mean platelet volume (MPV) has prognostic value for major adverse cardiovascular events (MACEs) in acute myocardial infarction without ST-segment elevation (NSTEMI) treated with clopidogrel. In this prospective observational cohort study, PDW, P-LCR, and MPV were determined on admission at the hospital and 24 h after clopidogrel treatment in 170 non-STEMI patients. MACEs were assessed over a one-year follow-up period. Using the Cox regression test, a decrease in PDW showed a significant association with the incidence of MACEs (odds ratio [OR] 0.82, 95% confidence interval [CI] 0.66–0.99, *p* ═ 0.049) and overall survival rate (OR 0.95, 95% CI ═ 0.91–0.99, *p* ═ 0.016). Patients with a decrease in PDW<9.9% had a higher incidence of MACEs (OR 0.42, 95% CI ═ 0.24–0.72, *p* ═ 0.002) and a lower survival rate (OR 0.32, 95% CI ═ 0.12–0.90, *p* ═ 0.03) than patients who had a decrease in PDW<9.9%. In the Kaplan–Meier analysis using log-rank test, patients who had a decrease in PDW<9.9% had an increased risk for MACEs (*p* ═ 0.002) and lethal outcomes (*p* ═ 0.002). However, a decrease in MPV or P-LCR did not have prognostic value. A decrease in PDW<9.9% measured 24 h after clopidogrel treatment in NSTEMI patients has good prognostic value for determining the short-term risks of MACEs, possibly providing a better risk stratification of those patients.

## Introduction

Acute coronary syndrome (ACS) is one of the most important causes of severe complications and death worldwide. ACS includes unstable angina pectoris, non-ST-elevation myocardial infarction (NSTEMI), and STEMI. After either NSTEMI or STEMI, major adverse cardiovascular events (MACEs), such as reinfarction, cardiac death, and requirement for target vessel revascularization (TVR), will occur. To improve the risk stratification of those patients, different inflammatory and hematological markers have been tested as predictive factors for MACEs after NSTEMI and STEMI. Among inflammatory markers, elevated C-reactive protein (CRP) has been proven to be the strongest independent factor in predicting overall lethal outcome, cardiac death, and MACEs in STEMI or NSTEMI patients after percutaneous coronary intervention (PCI) [1]. Platelets, as very important factors, are involved in the mechanisms of the development of cardiovascular diseases, such as atherosclerosis and ACS. Platelet indices as indicators of platelet activity have been demonstrated as markers of a prothrombotic state in cardiovascular diseases [2, 3].

Mean platelet volume (MPV) and platelet distribution width (PDW) values are higher in STEMI patients than in healthy individuals and correlates with the severity of STEMI [2]. Increased MPV is a strong independent predictive factor of short-term lethal outcome in STEMI, which independently correlates with long-term mortality in patients with STEMI and without STEMI [4].

Preprocedural values of thrombocytes and platelets indices PDW, platelet-large cell ratio (P-LCR), and MPV may predict MACE after PCI. Both thrombocytosis and thrombocytopenia on hospital admission in patients with ACS are associated with a long-term mortality rate [5].

Elevated MPV (≥9.6 fL) is a strong prognostic factor for MACE over a follow-up period of 1 year after an elective PCI procedure [6]. High MPV (≥11.7 fL), high PLC-R (≥31.8%), and high PDW (≥16 fL) are significantly associated with a higher mortality rate over 2 years of follow-up in STEMI and NSTEMI patients undergoing PCI [7]. A high preprocedural PDW value is a strong independent prognostic factor for the development of MACEs during hospitalization and long-term MACEs in ACS [8]. In patients with coronary bifurcation lesions undergoing PCI, high PDW is a good predictor of 1-year MACE [9]. A high MPV/PC (platelet count) ratio (≥0.055) has strong independent predictive value for long-term MACE in STEMI patients undergoing PCI [10].

In this research, we evaluated the prognostic value of decreased PDW, MPV, and P-LCR for MACEs in NSTEMI patients treated with clopidogrel.

## Materials and methods

### Patients

**Figure 1.**
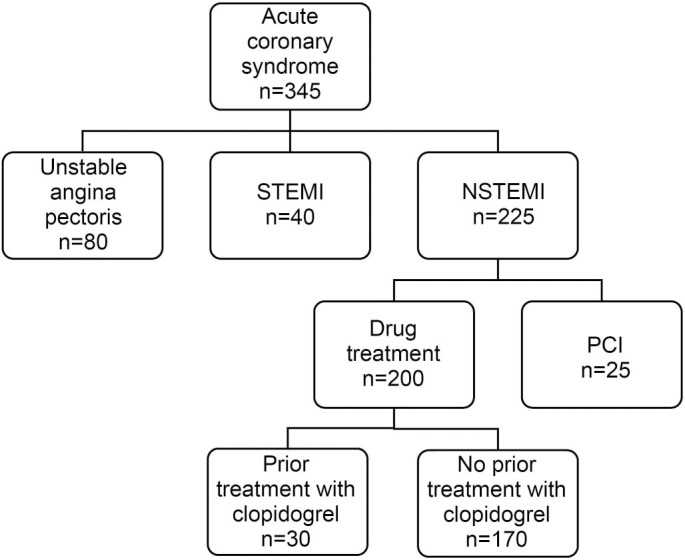
**A PRISMA chart of patient selection.** NSTEMI: Non-ST-elevation myocardial infarction; STEMI: ST-elevation myocardial infarction; PCI: Percutaneous coronary intervention.

This prospective observational cohort study included 170 NSTEMI patients who received conventional therapy, including clopidogrel (75 mg) and admitted to the Department of Cardiology, Clinic of Internal Diseases, University Clinical Center Tuzla, Bosnia and Herzegovina from January 1 to June 30, 2021. The cohort included only patients who had a decrease in MPV, PDW, and P-LCR values 24 h after clopidogrel treatment. The median of level of decrease used as cut-off value (it was 0.19 fL for MPV, and 9.9% for PDW and P-LCR). According to the median value of decrease in platelet indices 24 h after the first dose of clopidogrel, NSTEMI patients were split into: a) patients with a decrease in MPV ≤ 0.19 fL and patients with a decrease in MPV > 0.19 fL, b) patients with a PDW decrease <9.9% and patients with a PDW decrease ≥9.9%, and c) patients with a P-LCR decrease <9.9% and patients with a P-LCR decrease ≥9.9%. A PRIZMA chart of patient selection is shown in Figure 1.

In this study, the inclusion criteria for patients were: a) newly diagnosed NSTEMI, b) no prior clopidogrel treatment, and c) age >18. The exclusion criteria were: a) prior clopidogrel treatment for any indication, b) STEMI, c) stable coronary artery disease, d) prior NSTEMI, e) renal dysfunction, and f) hematological diseases.

### Laboratory tests

Venous blood samples were taken from all patients on admission and 24 h after the first dose of clopidogrel. Whole blood was immediately collected in a tube containing K2E (tetra acetic acid) anticoagulant. Blood samples were analyzed for platelet indices within 30 min using the Analyzer SYSMEX XN 100 (SYSMEX Corporation1-5-1 Wakinohama-Kaigandori, Chuo-Ku, Kobe 651-0073, Japan).

### Clinical follow-up

All NSTEMI patients underwent a follow-up for one year after admission to assess MACEs. MACEs included cardiac death, reinfarction, and the requirement for revascularization. The definitions for clinical outcomes (MACE) were according to International Classification of Diseases (ICD-10) [11]. Clinical follow-up was performed by telephone or through a patient’s examination.

### Ethical statament

The study was approved by the Ethical Committee of University Clinical Center Tuzla, Bosnia and Herzegovina, Decision No: 02-09/2-97-21.

### Statistical analysis

Continuous variables are expressed as the means ± SDs. They were analyzed by Student’s t-test. For parameters with a normal distribution, the Kolmogorov–Smirnoff test was used. Categorical variables are presented as frequencies (%) and were analyzed with the ꭓ^2^ test with Yates correction. Odds ratios (ORs) with 95% confidence intervals (CIs) are reported. The Kaplan–Meier test was used to estimate the survival rate. Cox regression analysis was used to determine the prognostic value of the variables. Differences between analyzed parameters were considered significant if the *p* value was <0.05.

## Results

The cohort in this prospective observational study included 170 NSTEMI patients (median age 68, IQR ═ 60–76, minimum 43, maximum 91) who received conventional therapy, including clopidogrel. There were 103 males (60.6%) and 67 females (39.4%). Clinical characteristics of patients with (*n* ═ 87) and without MACEs (*n* ═ 83), such as age, body mass index, hyperlipidemia, hypertension, diabetes mellitus, and current smoking did not differ significantly (Table 1).

**Table 1:** Clinical characteristics of patients with and without major adverse cardiovascular events (MACEs)

**Variable**	**All patients (*n* ═ 170)**	**Without MACEs (*n* ═ 83)**	**With MACEs (*n* ═ 87)**	***p* value**
Age (years)	68.1±2.3	66.5±6.4	68.4±7.8	0.085
Body mass index (kg/m^2^)	30.1±3.7	29.7±6.8	31.3±5.6	0.095
Diabetes mellitus, *n* (%)	71 (41.8)	34 (41.0)	37 (42.5)	0.836
Hyperlipidemia, *n* (%)	124 (72.9)	52 (62.7)	72 (82.8)	0.092
Current smoker, *n* (%)	85 (50)	36 (43.4)	49 (56.3)	0.096
Hypertension, *n* (%)	149 (87.6)	69 (83.1)	80 (92.0)	0.086

As a first step, we tested whether hematological parameters, including MPV, PDW, and P-LCR values, changed 24 h after clopidogrel treatment in NSTEMI patients. MPV (9.16 ± 1.45 vs 9.54 ± 1.55; *p* < 0.001), PDW (12.15 ± 2.09 vs 12.78 ± 2.41; *p* < 0.001), and PLC-R (29.61 ± 7.26 vs 30.76 ± 7.54; *p* < 0.001) values measured at 24 h after clopidogrel treatment were significantly decreased compared with those on the admission white blood cell count (10.34 ± 3.89 × 109/L vs 9.45 ± 3.70 × 109/L; *p* < 0.001), neutrophils (72.36 ± 12.34% vs 68.58 ± 13.38%; *p* < 0.001), eosinophils (1.55 ± 2.30% vs 1.13 ± 2.03%; *p* < 0.001), monocytes (8.61 ± 3.10% vs 7.61 ± 3.14%; *p* < 0.001), MCH (30.60 ± 2.44 vs 30.41 ± 2.30; *p* ═ 0.04), and PCT (0.03 ± 0.02 vs 0.0 ± 0.02; *p* < 0.01) were significantly increased at 24 h after the treatment. Values of other hematological parameters were not significantly changed (Table 2).

**Table 2:** Hematological parameters on admission and 24 h after clopidogrel treatment in patients with NSTEMI

	**On admission (mean±SD)**		**After 24 h (mean±SD)**		***p* value**
White blood cells (×10^9^/L)	9.45	±3.70	10.34	±3.89	<0.001
Red blood cells (×10^12^/L)	7.11	±34.56	4.42	±0.66	0.31
Hemoglobin (g/L)	135.34	±22.80	134.49	±21.03	0.30
Hematocrit	0.40	±0.06	0.40	±0.06	0.32
Platelets (×10^9^/L)	235.92	±78.04	232.94	±80.09	0.27
MCV	89.51	±5.72	89.61	±5.53	0.54
MCH	30.41	±2.30	30.60	±2.44	0.04
MCHC	339.73	±12.77	340.15	±11.97	0.55
MPV (fL)	9.54	±1.55	9.16	±1.45	<0.001
RDW-CV	14.11	±1.67	14.79	±9.19	0.33
RDW-SD	44.90	±5.52	45.09	±4.61	0.42
PCT	0.02	±0.02	0.03	±0.02	0.01
PDW	12.78	±2.41	12.15	±2.09	<0.001
P-LCR	30.76	±7.54	29.61	±7.26	<0.001
Monocytes (#)	0.78	±0.33	0.79	±0.60	0.72
Monocytes (%)	7.61	±3.14	8.61	±3.10	<0.001
Basophils (#)	0.04	±0.03	0.04	±0.03	0.16
Basophils (%)	0.41	±0.35	0.42	±0.29	0.50
Erythroblasts (#)	0.02	±0.09	0.01	±0.08	0.11
Erythroblasts (%)	0.13	±0.88	0.11	±0.86	0.11
Eosinophils (#)	0.10	±0.19	0.13	±0.24	0.05
Eosinophils (%)	1.13	±2.03	1.55	±2.40	<0.001
Lymphocytes (#)	1.71	±1.08	3.60	±23.73	0.30
Lymphocytes (%)	18.32	±10.21	21.19	±10.46	<0.001
Neutrophils (#)	7.28	±7.51	7.70	±3.74	0.41
Neutrophils (%)	68.58	±13.38	72.36	±12.34	<0.001

MCV: Mean cell volume; MPV: Mean platelet volume; PDW: Platelet distribution width; P-LCR: Platelet-large cell ratio; MCH: Mean cell hemoglobin; MCHC: Mean cell hemoglobin concentration; RDW-CV: Red cell distribution width-coefficient of variation; RDW-SD: Red cell distribution width-standard deviation; PCT: Platecrit; NSTEMI: Non-ST-elevation myocardial infarction.

Out of 170 patients, 89 (54%) had a decrease in MPV≤0.19 fL, and 81 (47%) had a decrease in MPV>0.19 fL. Fifty-six patients (32.9%) had a decrease in PDW ≥9.9%, and 114 (67.1%) had a decrease in PDW<9.9%. Forty-three (25.3%) patients had a decrease in P-LCR ≥9.9%, and 127 (74.7%) had a decrease in P-LCR < 9.9%. Clinical characteristics of patients did not significantly impact on a level of decrease in platelet indices (Table 3).

After myocardial infarction without ST-segment elevation, out of 170 patients, 87 (51.2%) had MACE, and 83 (48.8%) did not have MACE during the follow-up period. Out of 87 patients with MACE, 54 underwent PCI (coronary artery by-pass grafting *n* ═ 17 and drug-eluting stent implantation *n* ═ 37), and 25 died.

**Figure 2.**
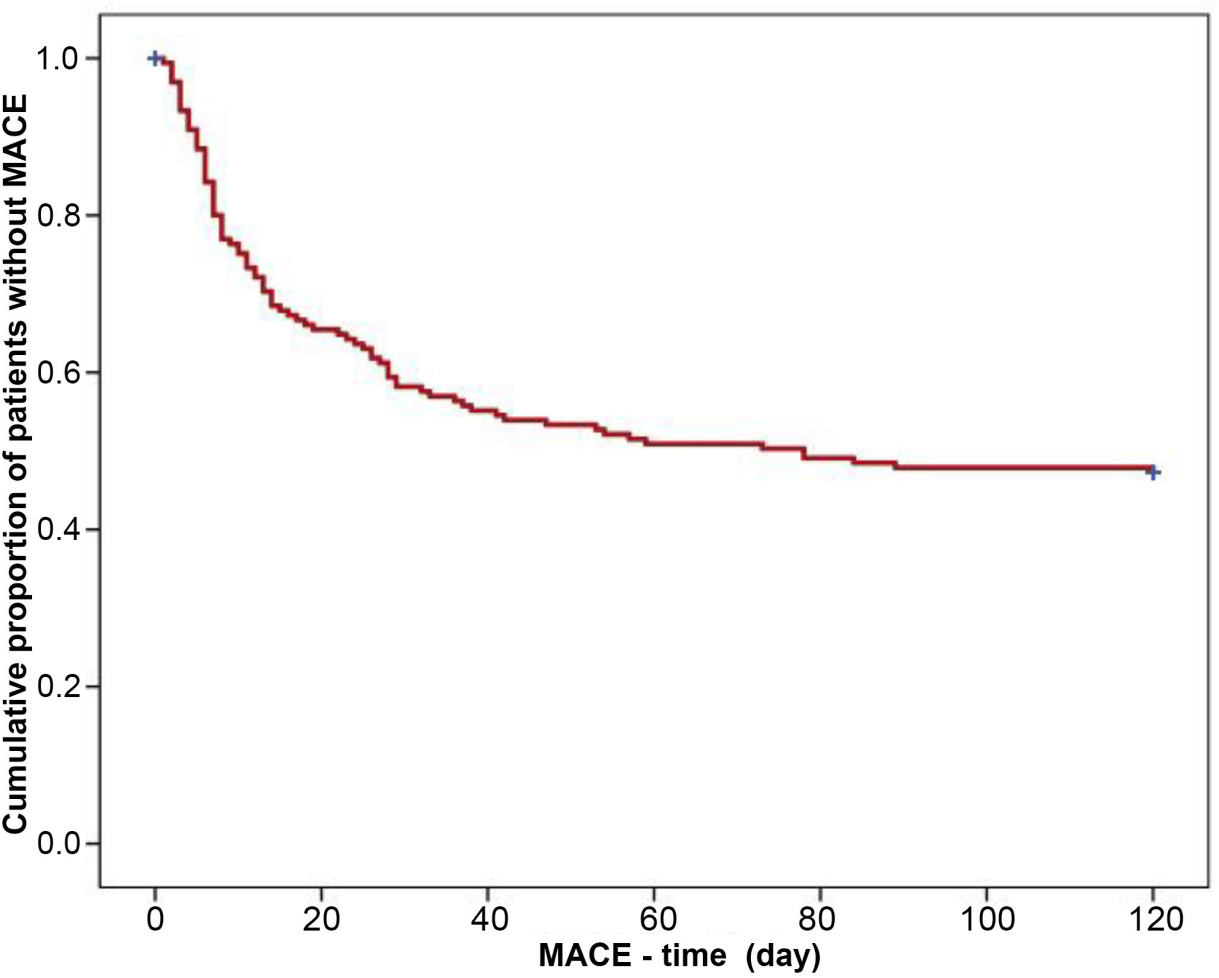
**Kaplan–Meier curves showing the overall mean time of the incidence of MACE in NSTEMI patients treated with clopidogrel.** *p* value is calculated by log-rank test. MACE: Major adverse cardiovascular event; NSTEMI: Non-ST-elevation myocardial infarction.

**Figure 3.**
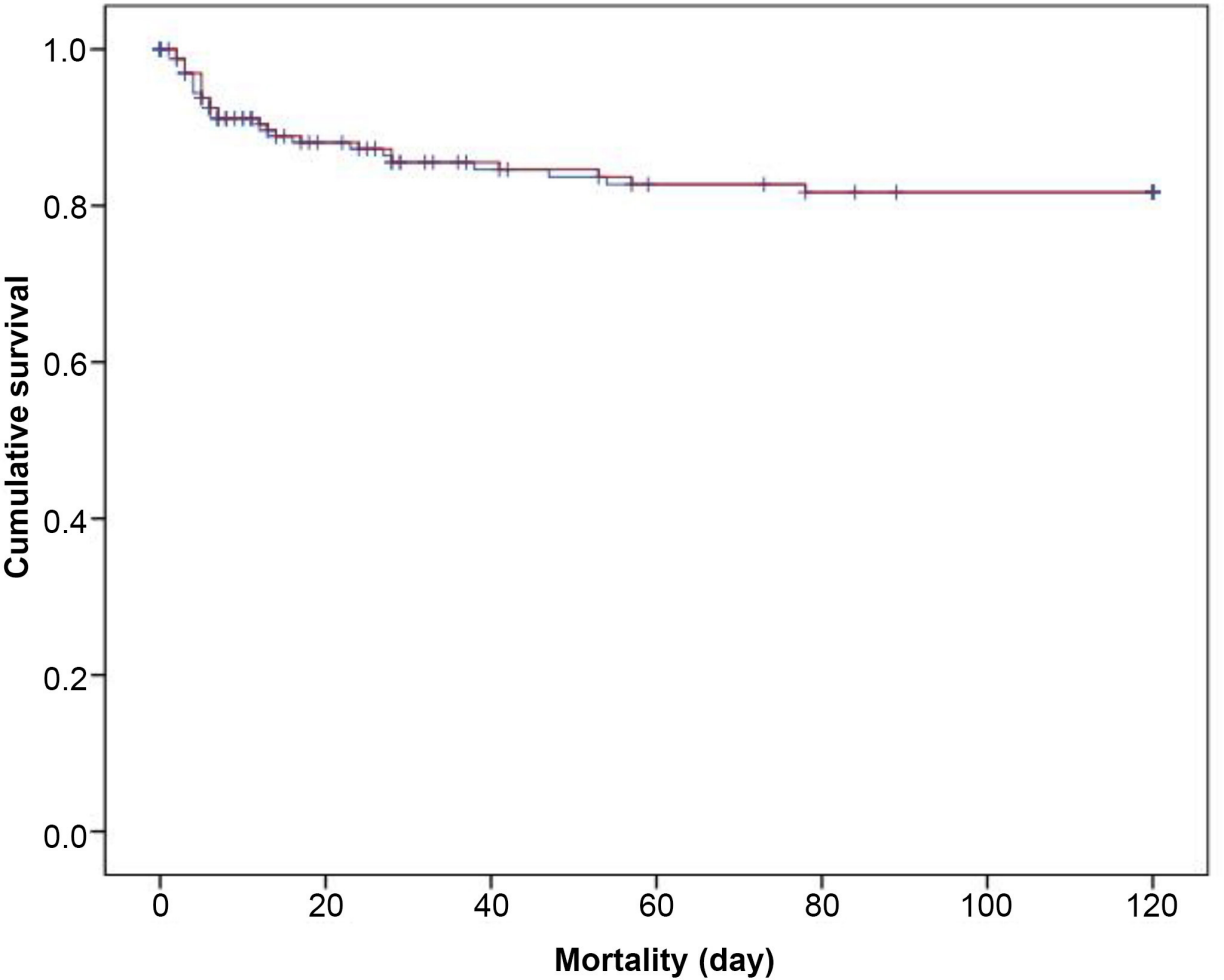
**Kaplan–Meier curves showing the overall mean time of the incidence of mortality in NSTEMI patients treated with clopidogrel.** *p* value is calculated by log-rank test. NSTEMI: Non-ST-elevation myocardial infarction.

The overall mean time of the incidence of MACE was 68 days (95% CI=60–76 days) (Figure 2), and the mean time of lethal outcome was 102 days (95% CI=95–108 days) (Figure 3).

**Figure 4.**
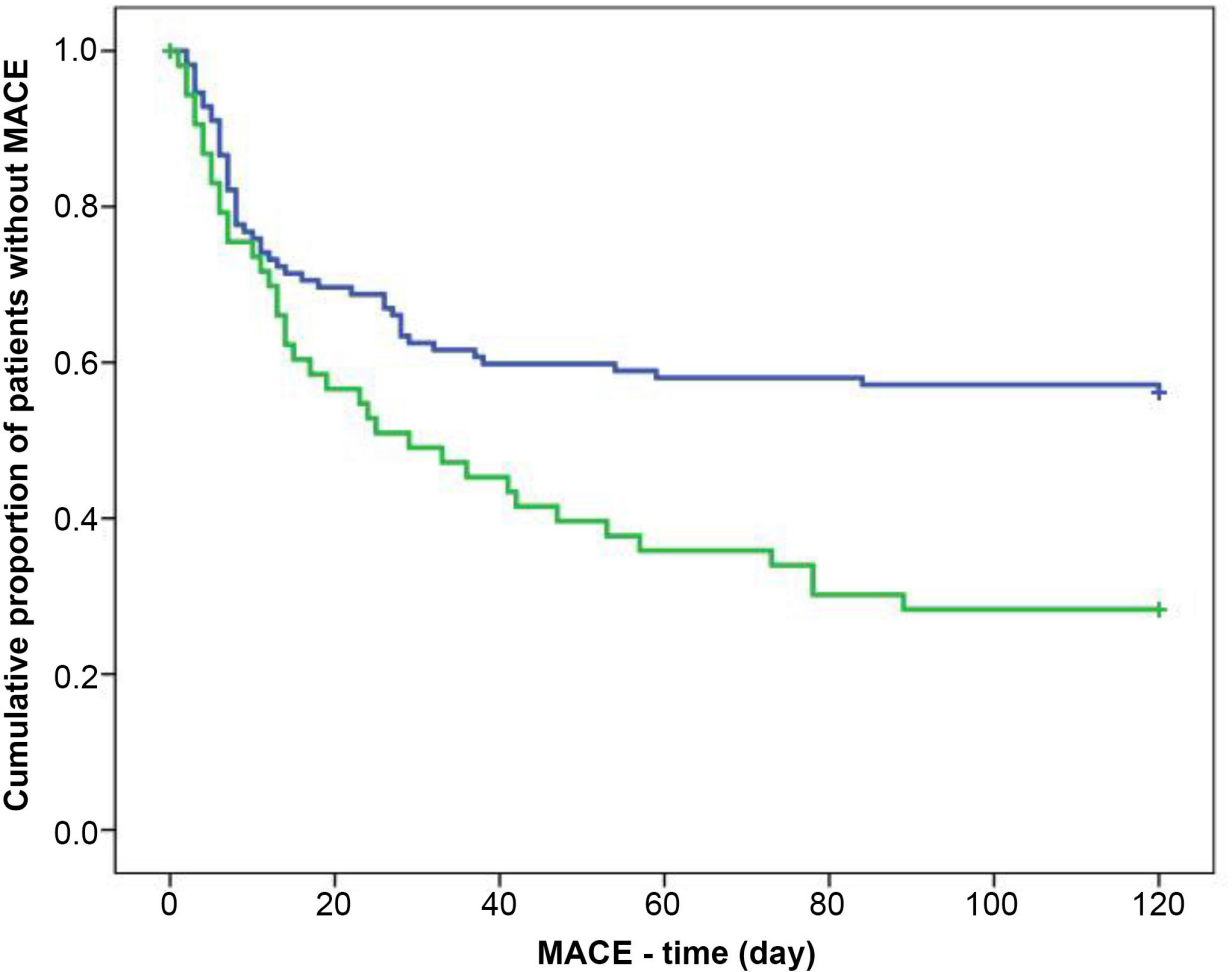
**Kaplan–Meier curves showing the incidence of MACE according to the decrease of PDW value in NSTEMI patients treated with clopidogrel (blue line—decrease of PDW≥9.9%, green line—decrease of PDW <9.9%).** *p* value is calculated by log-rank test. MACE: Major adverse cardiovascular event; PDW: Platelet distribution width; NSTEMI: Non-ST-elevation myocardial infarction.

Using Cox regression analysis, we found that only the level of decrease in PDW showed a statistically strong association with the incidence of MACEs (OR 0.82, 95% CI 0.66–0.99, *p* ═ 0.049). A decrease in PDW<9.9% increased the risk for MACEs (OR 0.42, 95% CI 0.24–0.72, *p* ═ 0.002). However, MPV or PLC-R did not have prognostic value. The Kaplan–Meier analysis showed that patients who had a decrease in PDW<9.9% had an increased risk for MACEs (*p* ═ 0.002) (Figure 4).

**Figure 5.**
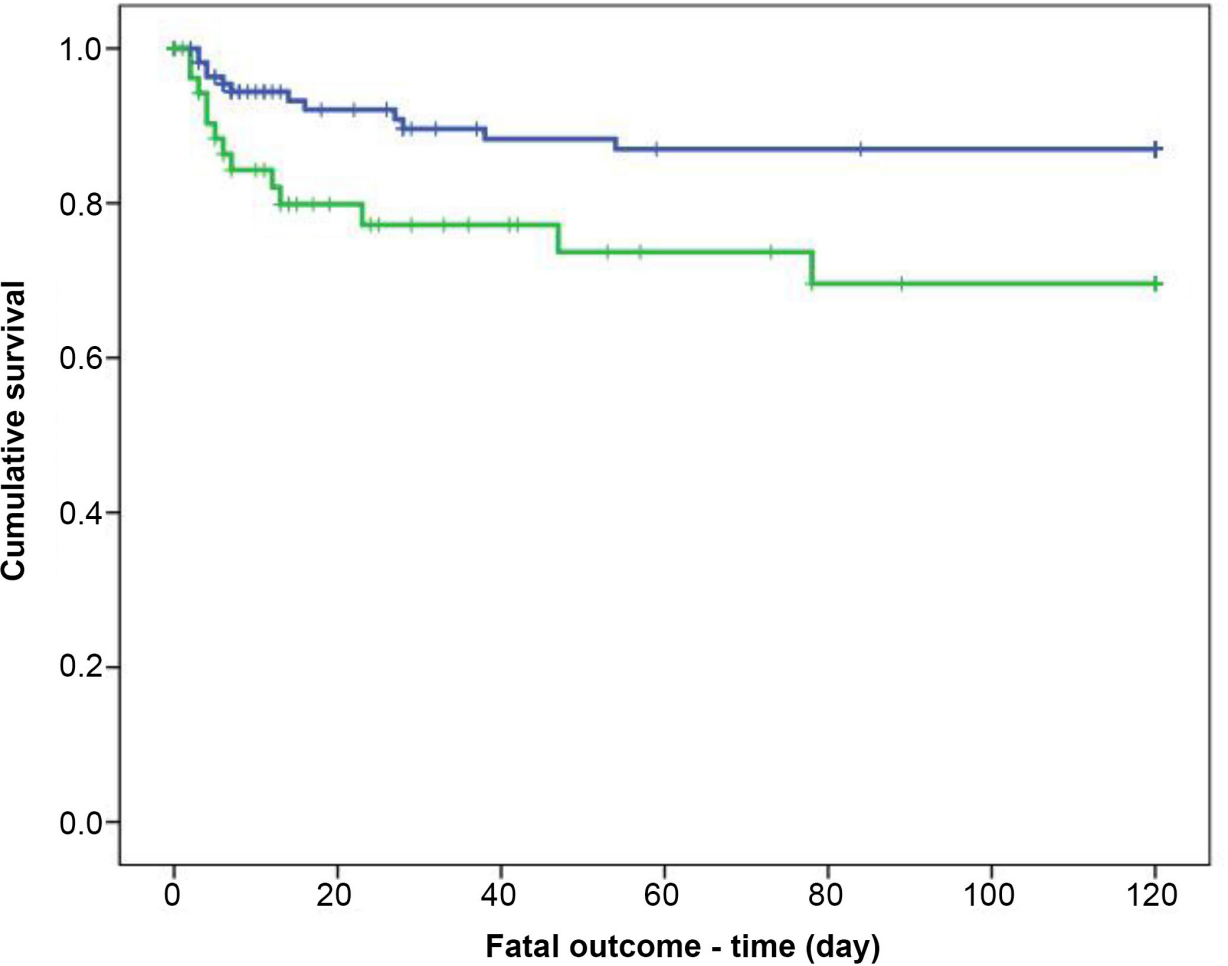
**Kaplan–Meier curves showing the incidence of mortality according to the decrease of PDW value in NSTEMI patients treated with clopidogrel (blue line—decrease of PDW≥9.9%, green line—decrease of PDW <9.9%).** *p* value is calculated by log-rank test. PDW: Platelet distribution width; NSTEMI: Non-ST-elevation myocardial infarction.

Cox regression analysis showed that only a decrease in PDW was independently associated with the overall survival rate (OR 0.95, 95% CI=0.91–0.99, *p* ═ 0.016). Patients with a decrease in PDW≥9.9% had a higher survival rate than patients with a decrease in PDW<9.9% (OR 0.32, 95% CI ═ 0.12–0.90, *p* ═ 0.03). In the Kaplan–Meier analysis, patients with a decrease in PDW<9.9% had a higher risk for a lethal outcome (*p* ═ 0.002) (Figure 5).

**Table 3:** Clinical characteristics of patients stratified according to the median of level of decrease in MPV, PDW, and P-LCR measured 24 h after clopidogrel treatment

**Variable**	**MPV**			**PDW**			**P-LCR**		
	**≤0.19 fL (*n* ═ 89)**	**>0.19 fL (*n* ═ 81)**	***p value***	**<9.9% (*n* ═ 114)**	**≥9.9% (*n* ═ 56)**	***p value***	**<9.9% (*n* ═ 127)**	**≥9.9% (*n* ═ 43)**	***p value***
Age (years)	68.6±4.9	66.8±7.5	0.064	69.1±9.2	67.8±6.7	0.348	68.9±7.6	66.3±8.3	0.060
Body mass index (kg/m^2^)	30.9±3.5	29.8±4.7	0.084	31.2±7.8	29.4±9.3	0.187	30.7±8.2	28.7±6.9	0.153
Diabetes mellitus, *n* (%)	40 (44.9)	31 (38.3)	0.381	52 (45.6)	19 (33.9)	0.164	56 (44.1)	15 (34.9)	0.311
Hyperlipidemia, *n* (%)	68 (76.4)	56 (69.1)	0.292	89 (78.1)	35 (62.5)	0.053	93 (73.2)	31 (72.1)	0.143
Current smoker, *n* (%)	46 (51.7)	39 (48.1)	0.646	62 (54.4)	23 (41.1)	0.122	69 (54.3)	16 (37.2)	0.077
Hypertension, *n* (%)	82 (92.1)	67 (82.7)	0.067	104 (91.2)	45 (80.4)	0.079	111 (87.4)	38 (88,4)	0.209

MPV: Mean platelet volume; PDW: Platelet distribution width; P-LCR: Platelet-large cell ratio.

## Discussion

The inflammatory response and prothrombotic state are involved in the development of ACS. Markers of inflammation and thrombotic activity may reflect the severity of ACS and may predict the development of MACE after certain types of ACS treatment, such as PCI or drug therapy. Increased platelet activity leads to increased thrombotic activity and increases the risk for MACE in patients with ACS. It correlates with increased values of platelet indices: MPV, PDW, and PLC-R. Clopidogrel decreases the risk for MACEs by lowering platelet activity, but the novel P2Y12 inhibitors ticagrelor and prasugrel reduce long-term mortality and MACE compared to clopidogrel in patients with ACS [12].

Increased MPV has been associated with MACEs in patients with STEMI undergoing PCI [2, 7, 10, 13, 14] and in patients without STEMI [15, 16]. High MPV has demonstrated a strong and independent association with an increased risk of plaque rupture in NSTEMI patients [17]. Nevertheless, elevated MPV did not predict in-hospital mortality in NSTEMI patients [4]. In our study, we found that MPV (9.16 ± 1.45 vs 9.54 ± 1.55; *p* < 0.001) values measured at 24 h after clopidogrel treatment in the patients without STEMI were significantly decreased compared with those on admission, but MPV remained high (>9.0 fL). Out of 170 patients, 89 (54%) had a decrease in MPV≤0.19 fL and 81 (47%) had a decrease in MPV>0.19 fL; there was no significant difference. Using Cox regression and Kaplan–Meier analyses, MPV did not have prognostic value for MACEs. This could be due to an insufficient decrease in MPV and might be because of the resistance to clopidogrel. Up to our knowledge, there is no similar study investigating the dynamic changes of MPV within 24 h after clopidogrel treatment in NSTEMI patients. There is only one study about the dynamic changes of MPV and cardiac function in patients with acute myocardial infarction (AMI) who underwent PCI. Wang et al. [18] in this study reported that dynamic changes of MPV occurred over an acute phase of AMI and MPV was high in patients with high Killip Class, suggesting a predictive value of MPV in ventricular dysfunction and clinical outcome in AMI. Increased PDW for 1 fL (13.8%) and plateletcrit for 1% are associated with STEMI in young patients [3]. The PDW value was significantly greater in NSTEMI patients who had inadequate coronary collateral development (CCD) than in those with adequate CCD [19]. High PDW is strongly associated with STEMI and correlates with the severity of STEMI [2]. High PDW upon arrival to a hospital is a strong independent prognostic factor for MACE in patients with or without STEMI treated by PCI in short and long follow-up periods [7–9, 20, 21]. In this study, the decrease of PDW was independently associated with the incidence of MACEs (OR 0.82, 95% CI 0.66–0.99, *p* ═ 0.049) and overall survival rate (OR 0.95, 95% CI=0.91–0.99, *p* ═ 0.016). Patients with decrease of PDW<9.9% had higher incidence of MACEs (OR 0.42, 95% CI 0.24–0.72, *p* ═ 0.002) and lower survival rate (OR 0.32, 95% CI ═ 0.12–0.90, *p* ═ 0.03) than patients with decrease of PDW≥9.9%. Using the Kaplan–Meier test, patients who had a decrease of PDW<9.9% showed increased risk for MACEs (*p* ═ 0.002) and lethal outcome (*p* ═ 0.002). Our results are consistent with those mentioned above regarding the association of the PDW value with MACEs.

High P-LCR on admission is a strong predictive factor for MACEs in patients with or without STEMI undergoing PCI [7]. In the study by Małyszczak et al. [5], PDW and P-LCR were not associated with MACEs in patients with ACS. In our study, PLC-R did not have prognostic value for MACEs in patients without STEMI treated with clopidogrel.

Some patients with ACS develop resistance to clopidogrel, which decreases its antiplatelet activity, leading to less efficiency in preventing the development of MACEs [22, 23]. In our study, clopidogrel significantly changed only PDW and was a strong independent predictor for MACE, but MPV and P-LCR did not have prognostic value. This seems to be due to partial resistance to clopidogrel.

Our study has two limitations: a) a relatively small number of patients were enrolled in the study and b) it was conducted in a single center.

## Conclusion

In conclusion, the level of decrease in PDW<9.9% measured 24 h after clopidogrel treatment in NSTEMI patients has good prognostic value for determining the short-term risks of MACEs and possibly providing a better risk stratification of those patients.
